# The Effect of Heat Treatment and Soaking in PBS Solution on the Strength Properties of PEEK Intended for Use in Orthopedics

**DOI:** 10.3390/ma19050875

**Published:** 2026-02-26

**Authors:** Gabriela Wielgus, Wojciech Kajzer, Jan Juszczyk, Aleksandra Kopica, Anna Ziębowicz, Anita Kajzer

**Affiliations:** 1 Department of Biomaterials and Medical Devices Engineering, Faculty of Biomedical Engineering, Silesian University of Technology, Roosevelta 40 Street, 41-800 Zabrze, Poland; ak306924@student.polsl.pl (A.K.); anna.ziebowicz@polsl.pl (A.Z.); anita.kajzer@polsl.pl (A.K.); 2 Department of Biomechatronics, Faculty of Biomedical Engineering, Silesian University of Technology, Roosevelta 40 Street, 41-800 Zabrze, Poland; wojciech.kajzer@polsl.pl; 3 Department of Medical Informatics and Artificial Intelligence, Faculty of Biomedical Engineering, Silesian University of Technology, Roosevelta 40 Street, 41-800 Zabrze, Poland; jan.juszczyk@polsl.pl

**Keywords:** non-degradable PEEK polymer, additive technologies, strength testing, heat treatment, soaking in PBS solution, personalized medicine

## Abstract

PEEK (polyetheretherketone) is a semi-crystalline thermoplastic polymer which, thanks to its excellent mechanical properties, chemical resistance, and high biocompatibility, is widely used in medicine, especially in biomedical engineering. The dynamic development of additive technologies, especially FFF (Fused Filament Fabrication), has enabled the production of personalized medical implants from PEEK, such as skull implants, dental implant components, and orthopedic implants like spine, knee and hip implants. Therefore, the aim of the study was to evaluate the polymer as an alternative material for orthopedic applications and to analyze the effect of annealing and soaking in PBS solution on its strength properties. Heat treatment improves the strength properties of the material. On the other hand, prolonged soaking in PBS solution, which simulates physiological conditions, can lead to changes in the interlayer bonds of the filament layers, which in turn affects the strength properties of the material.

## 1. Introduction

Modern biomaterials are an important element of technological progress in medicine, contributing to the development of modern solutions for the stabilization of fractures/joint dislocations, which in turn leads to improved treatment effectiveness and better quality of healthcare [1,2,3,4]. The physicochemical and mechanical properties of polymer materials such as PMMA (polymethyl methacrylate), PEEK (polyetheretherketone), and PLA (polylactic acid) enable the production of personalized implants using additive technologies such as SLS (selective laser sintering) and FFF (fused filament fabrication) [5,6,7,8,9,10].

In recent years, PEEK has been increasingly used in the production of orthopedic and dental implants as an alternative to titanium alloys such as Ti6Al4V and Ti6Al7Nb [11,12,13]. One of the important features of this polymer is its similar longitudinal elastic modulus (for PEEK E = 4400 MPa, for cortical bone E = 17,700 MPa), which allows for the more accurate representation of the natural biomechanics of the musculoskeletal system compared to titanium alloys. The basic mechanical properties of titanium alloys and PEEK are summarized in Table 1.

**Figure 1:**
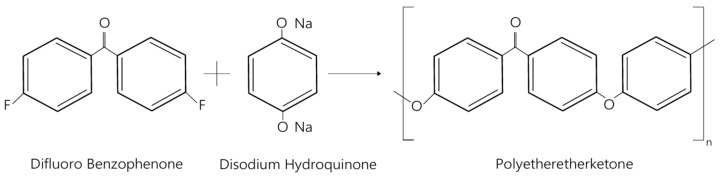
Structure of polyetheretherketone. Reprinted from Ref. [9].

PEEK belongs to a broad group of thermostable ketone polymers known as polyaryletherketones (PAEK). Polymers from this family are characterized by diverse chemical compositions, which are related to varying proportions of ether and ketone groups in the molecular chain [18,19]. PEEK synthesis is most often based on the reaction of difluorobenzophenone with disodium hydroquinone in a polycondensation process (Figure 1).

Polyetheretherketone is a high-temperature thermoplastic polymer with a semi-crystalline structure, whose performance properties can be controlled by appropriately selected heat treatment. It is characterized by excellent mechanical properties, high thermal resistance (operating temperature 250–260 °C, processing temperature 370–400 °C), and excellent chemical resistance, including hydrolysis resistance [18,22].

The polyetheretherketone filament processed using FFF technology is mostly used in the production of customized orthopedic implants. An alternative approach involves using PEEK granules, which, after being ground into powder, can be modified by adding hydroxyapatite. The composite obtained in this way is a material with enhanced bioactive properties, which promotes the improved osseointegration of the implant with bone tissue [23,24,25,26,27,28]. To ensure adequate interlayer adhesion and minimize structural defects, it is crucial to select the right 3D printing parameters, which directly affect the strength properties and microstructure of the manufactured components. In the case of PEEK printing, one of the most important technological factors is the extrusion speed, which determines the degree of layer bonding and the angle of the samples to be printed. Samples printed in a 0° orientation exhibit a flexural strength of 122.53 MPa, while samples printed in a 90° orientation achieve a value of 156.84 MPa [23]. The most favorable strength properties are obtained at a standard extrusion speed of 1.0×. Too low a printing speed (0.8×) reduces the degree of material filling, resulting in a poor bond between the individual layers of material. Tensile strength drops from 69.35 MPa to 31.78 MPa and bending strength drops from 122.53 MPa to 56.48 MPa. On the other hand, increasing the speed above the standard results in insufficient filling and poor interlayer bonding [23]. The second important parameter affecting the strength of PEEK products is the fill angle. In FFF technology, the standard print angle is ± 45°, but based on data from the literature [23], the authors indicate that changing this angle by ± 10° leads to a significant improvement in the strength properties of the material. This effect results from a more favorable arrangement of fibers in the direction of the force, which reduces the concentration of shear stresses and promotes a more homogeneous internal structure of the print. The orientation of the samples for printing also has a particular impact on strength. Printing at a 90° angle to the substrate, i.e., with the material layers arranged along the direction of the load, increases the flexural strength by up to 28% compared to printing at a 0° angle [23]. Also, to assess changes in strength properties under conditions like the physiological environment, PEEK samples are subjected to a process of soaking in a PBS (phosphate-buffered saline) solution for a period of 28 days at a temperature of 37 °C. This process simulates the effect of body fluids on the material, allowing for analysis of its chemical stability and the impact of the environment on its structure and interlayer integrity [24,25,26,27,28,29].

In summary, it can be concluded that the results of the conducted research relate exclusively to determining the impact of individual parameters on the strength properties of PEEK, while there is still no research that considers the angle of the inclination of the samples for printing, the heating process, and immersion in PBS to simulate body fluids.

Therefore, the main objective of the publication was to evaluate the impact of sample orientation (0° and 90° angles) when printing using FFF technology, as well as the impact of material annealing using strength properties. It should be noted that the samples intended for static tensile testing were printed exclusively in the 0° orientation. This is due to the requirements related to the strength tests and the need to ensure the correct and stable mounting of the samples in the strength testing machine clamps. Additionally, the samples were immersed in a PBS solution to determine the effect of an environment simulating body fluids on their strength properties. This issue is of significant importance in the context of biomaterial applications in implantology.

## 2. Materials and Methods

**Figure 2:**
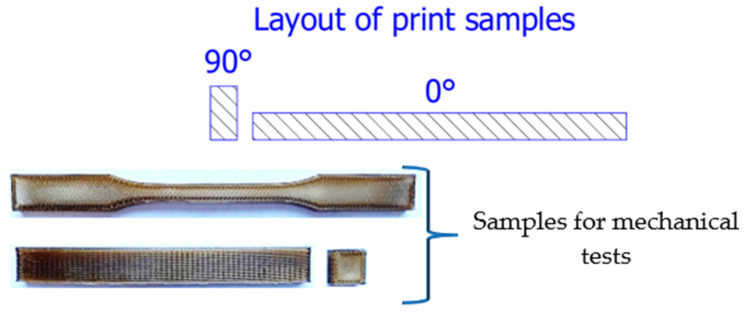
Arrangement of samples for 3D printing and model of samples for strength testing.

The “PEEK Natural” filament from 3DGENCE was used to produce samples using FFF technology [30,31]. The samples were intended for strength testing, which included static tensile, flexural, and compressive tests. The sample models were designed in Autodesk Inventor Professional 2024 in accordance with the applicable normative recommendations: ISO 178, ISO-527-2, and ISO 604 [32,33,34]. The samples for strength testing were printed on an Industry F420 printer in a vertical position (sample orientation during printing 90°) and horizontal position (sample orientation during printing 0°). The sample models and the sample arrangement for testing are shown in Figure 2, while the printing parameters are presented in Table 2.

To obtain a semi-crystalline structure, the sample underwent the heating process in a 3DGENCE furnace for 16 h at a temperature of 156 °C. The samples were placed in sand, as this material has low thermal conductivity and good thermal insulation properties. This allows for the gradual and uniform heating and cooling of the PEEK sample, preventing thermal shock and associated deformation. The final stage of sample preparation for testing was the immersion of the samples in a PBS solution (pH 7.4) for 28 days at 37 °C in a Q-CELL laboratory incubator, which simulated physiological conditions. A detailed breakdown of the samples for strength testing is presented in Table 3.

Machine learning can accelerate the analysis of strength test results, contributing to the optimization of the research process. ML methods, which are among the key areas of artificial intelligence, enable the effective discovery and optimization of materials with desired properties based on experimental or theoretical data [35]. However, a significant limitation of the application of machine learning in the context of the research presented in this article is the lack of real data that can be used for machine learning. In this article, authors focused on physical research, but authors know that the use of machine learning methods and in silico experiments can accelerate the research process and analysis.

### 2.1. X-Ray Diffraction 

The X-ray diffraction studies were performed using D8 Advance diffractometer (Bruker AXS, Karlsruhe, Germany) [35,36,37,38], equipped with copper tube (working at 40 kV and 40 mA) and a LYNXEYE XE-T linear detector (Bruker AXS, Karlsruhe, Germany). The scans were performed in the range of 5° to 80° 2Θ, with a step of 0.02°. The crystallinity of polymer was calculated using peak decomposition method.

### 2.2. Surface Roughness

Topography and surface roughness tests were performed on samples printed at a 0° orientation using a Leica Microsystems profilometer with LeicaSCAN 6.6 software (Laica Microsystems, Wetzlar, Germany). The results were processed using Mountains Imaging Topography 10 software. During the tests, the parameters *Sa* (arithmetic mean height) and *Ra* (arithmetic mean profile ordinates) were determined in accordance with the applicable ISO standards for surface topography assessment [39,40]. The results of the research were presented in the form of topographic maps and histograms. The area of each sample tested was 0.48 mm^2^.

### 2.3. Mechanical Tests

Static tests: Tensile, compressive and bending tests were performed on an MTS Criterion model 45 (MTS Systems Corporation, Eden Prairie, MN, USA) testing machine (with MTS TestSuite TW Elite). Each test consisted of three measurement trials. The results of the static strength tests are presented in the form of force–displacement graphs and tables showing the maximum tensile, compressive and flexural forces, as well as the corresponding material strength values.

#### 2.3.1. Tensile Strength Test

**Figure 3:**
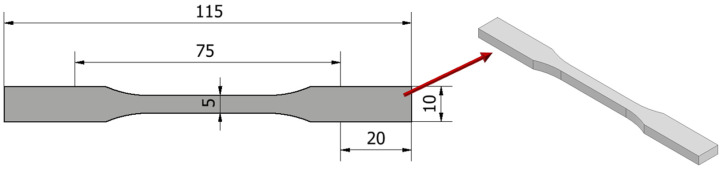
Sample model for static tensile testing.

The static tensile test was performed in accordance with the recommendations of the standard ISO-527-2 [33] at a tensile speed of 1 mm/min. Standardized samples in the form of paddles were used for the tests (additionally 20 mm was added to each side of the “paddle” in order to fix the sample correctly in the holder), as shown un Figure 3. For each group of samples, the maximum tensile force—F_m_ [N] and tensile strength R_m_ [MPa] were determined.

#### 2.3.2. Compressive Strength Test

**Figure 4:**
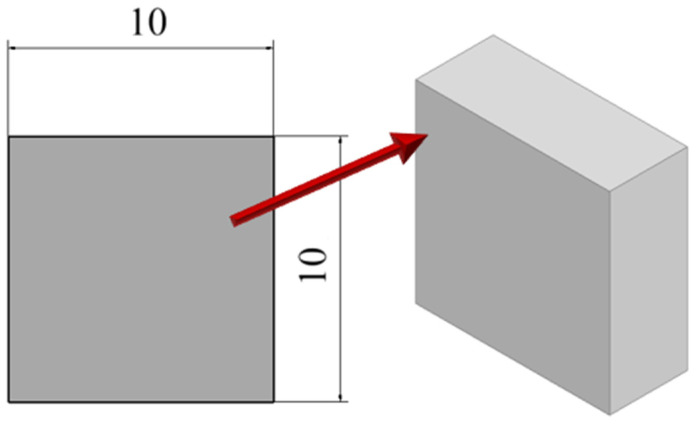
Sample model for static compression testing.

The static compressive test was performed in accordance with the recommendations of ISO 604 [34] at a compression speed of 1 mm/min ± 20%. Standardized samples in the form of a right prism (rectangular prism) were used for the tests, as shown in Figure 4. Based on the results obtained, the maximum compressive force, P_c_ [N], and compressive strength, R_c_ [MPa], were determined.

#### 2.3.3. Bending Strength Test

**Figure 5:**
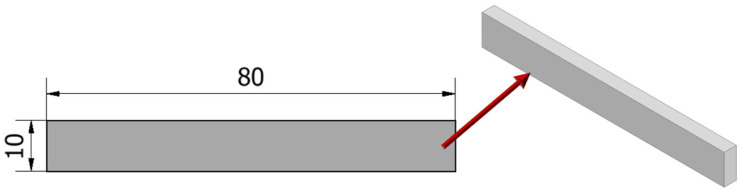
Sample model for static bending test.

The static bending test was performed in accordance with the recommendations of the standard: ISO 178 [32] with a bending speed of 1 mm/min and a support spacing of 60 mm. Standardized samples in the form of a right prism rectangular cross-section were used for the tests, as shown in Figure 5. The test was carried out until the sample broke or until a maximum deformation of 5% was reached. Based on the graphs for individual sample groups, the maximum bending force, F_g_ [N], and bending strength, R_g_ [MPa], were determined. In addition, the deflection value, *f* [mm], was determined.

#### 2.3.4. Hardness Measurement

Hardness was measured using a Shore D hardness tester (SAUTER, Balingen, Germany; cone penetrator). Five tests were performed on each sample. Measurements were taken at least 9 mm from the edge of the sample, and the results were read 3 s after applying pressure, in accordance with ISO 868:2003 [41].

### 2.4. Macroscopic Observations

Macroscopic observations of samples after strength testing were performed using a Leica DVM6 User Manual digital microscope (Laica Microsystems, Wetzlar, Germany). The observations were made at 70× magnification. In addition, based on microscopic observations at 250× magnification, measurements of the distance between adjacent filament layers were made to assess the homogeneity of the structure and the quality of interlayer bonds. The presentation of the results was limited to samples subjected to static tensile testing, as the greatest differences in the strength test results were observed in their case, which could also be the result of different distances between the filament layers for individual sample groups.

### 2.5. Computed Tomography

The examination was performed using a Procon X-Ray (Sarstedt, Nümbrecht, Germany) microtomography scanner with the following parameters: voltage, 70 kV; exposure, 250 microamperesecond. The reconstruction was performed using X-aid software (MITOS GmbH, Munich, Germany) with the FDK reconstruction algorithm. The final voxel size was 0.015 mm. Samples intended for static compressive testing were subjected to tomographic analysis. The selection of this group of samples was dictated by the limited working space of the tomograph. Measurements of the distance between adjacent fiber layers were performed using Slicer 5.8.1 software.

### 2.6. Statistical Analysis

In order to identify statistically significant differences for *p* < 0.05 between the studied groups, one-way analysis of variance (ANOVA) was performed.

## 3. Results and Discussion

### 3.1. X-Ray Diffraction—Results and Discussion

**Figure 6:**
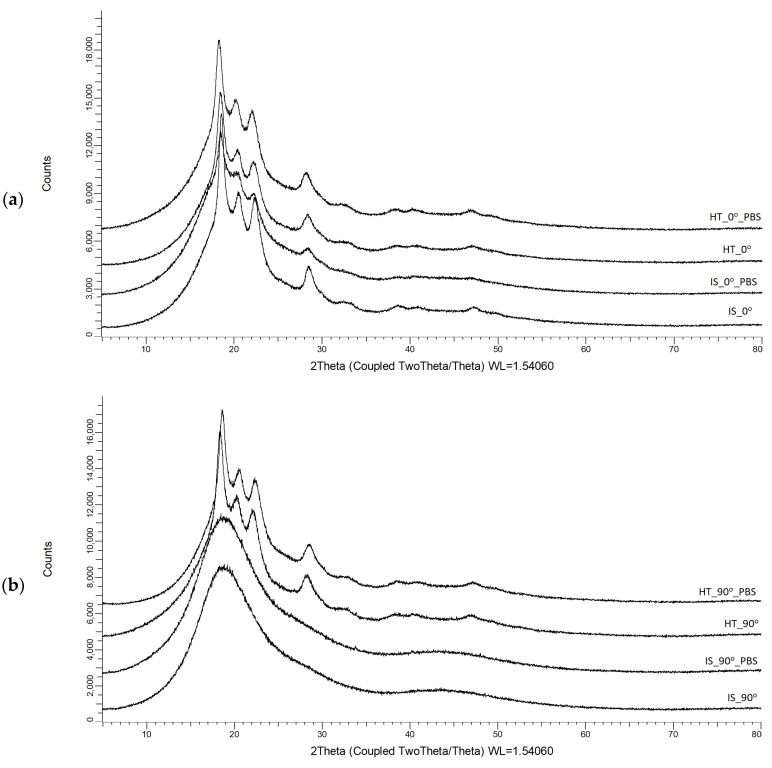
X-ray diffraction patterns of samples printed with (**a**) 0° and (**b**) 90° orientations.

X-ray diffraction patterns of samples printed with 0° (a) and 90° (b) orientations are presented in Figure 6.

The patterns obtained are given in Figure 6, while the calculated crystallinity is listed in Table 4. One can notice that samples printed at 90° show an amorphous nature from the beginning, while samples printed at 0° show a crystallinity of about 30%. This phenomenon is related to the orientation of the samples in the additive printing process. Samples printed at a 90° orientation had a smaller contact area with the work platform, while samples at a 0° orientation had a larger surface area adjacent to the work platform, which was additionally annealing. This arrangement of the samples may have contributed to a more uniform temperature distribution and slower cooling of the material, which in turn may have led to a reduction in the amorphous phase in the structure of the samples in the initial state IS_0. Heat treatment results in a significant modification in the organization’s structure in crystallinity, while after immersion in PBS, crystallinity slightly increases in all cases. It is a result of the slow dissolving of amorphous phases in the solution.

### 3.2. Surface Roughness–Results and Discussion

**Figure 7:**
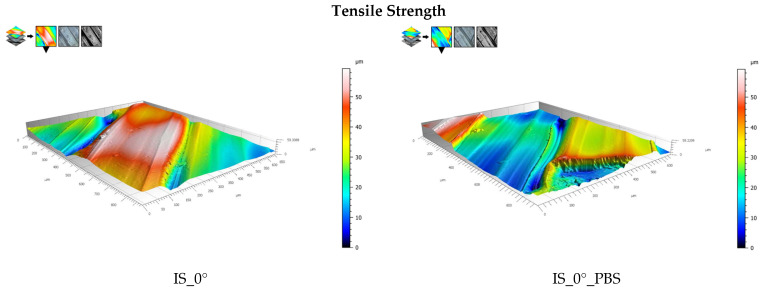

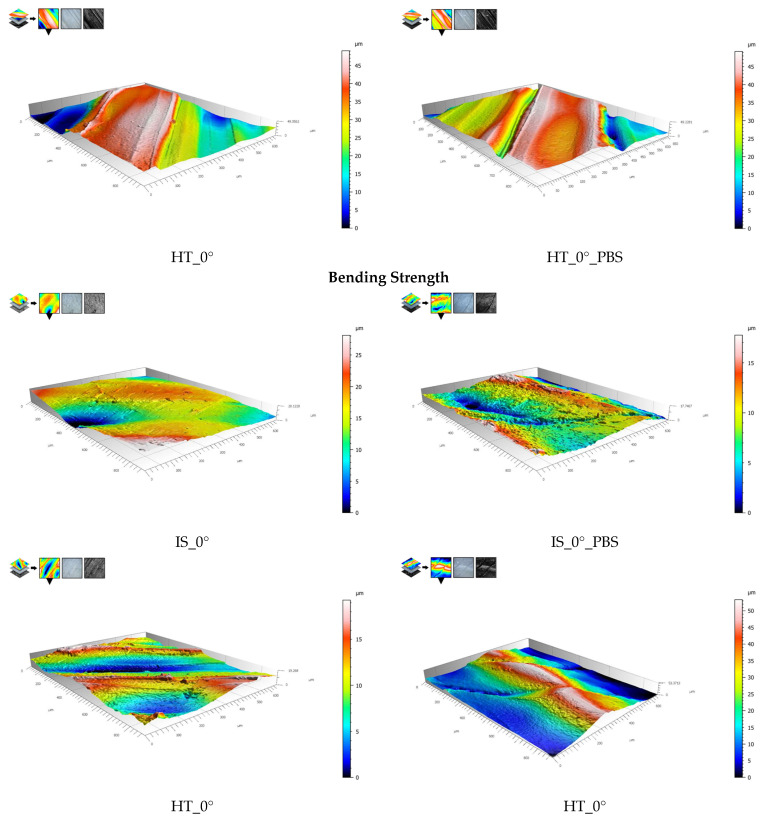

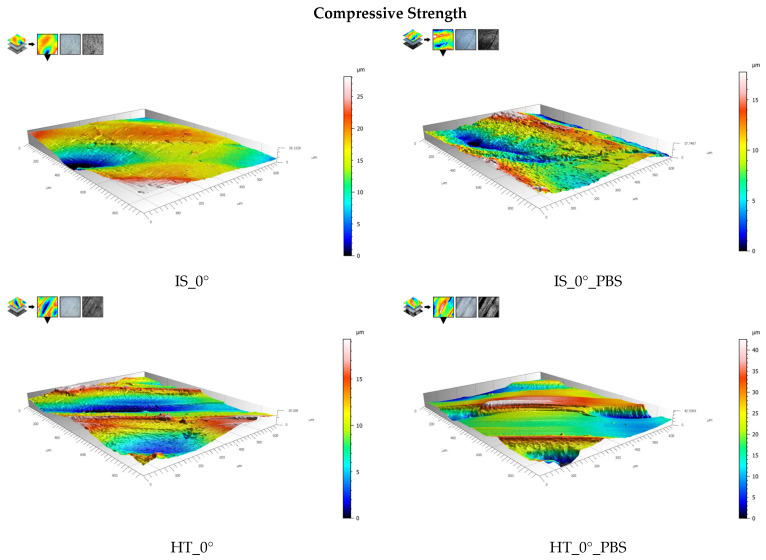
Topography maps of samples for tensile strength, bending, and compressive testing.

**Figure 8:**
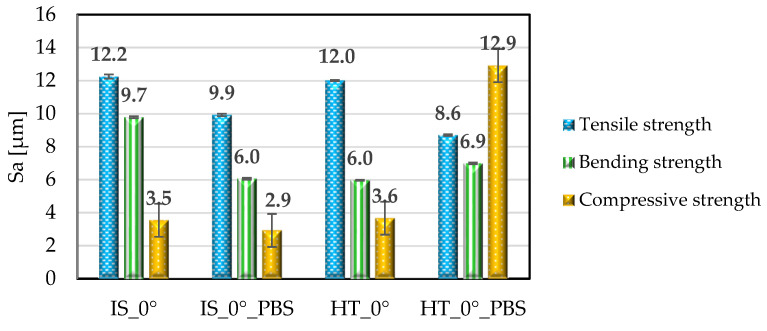
Comparison of the Sa parameter for sample surfaces. Error bars represent standard deviation.

**Figure 9:**
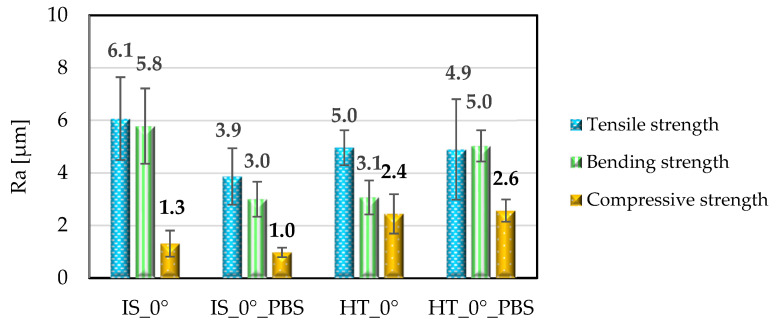
Comparison of the Ra parameter for sample surfaces. Error bars represent stndard deviation.

Examples of surface topography maps of the samples are shown in Figure 7, while the roughness measurement results are presented in Figure 8 and Figure 9.

The data analysis revealed that the surfaces of the samples in the initial state IS_0° were characterized by the highest value of the *Sa* parameter compared to samples from the same group that were subjected to heat treatment or soaked in PBS solution. The greatest changes were observed for samples soaked in PBS solution; this applies to samples intended for static tensile testing. In the case of samples for static compression testing, it can be observed that for samples in the initial state, the *Sa* parameter had the highest value. For the same group of samples soaked in PBS solution, the value of this parameter decreased from 9.6 μm to 6.0 μm. The same result was observed for annealing samples. In contrast, the surfaces of the samples for the static bending test were characterized by an *Sa* parameter value ranging from 2.9 to 3.6 μm, which was also confirmed by ANOVA statistical analysis (*p* < 0.05). The highest *Sa* parameter value was observed for the sample from the HT_0°_PBS group. This value increased to 12.9 μm. In the case of the *Ra* parameter, the highest values were recorded for samples for static tensile testing. Samples for static bending testing showed a similar range of *Ra* values, while the lowest values were recorded for all groups of samples for static compression testing, ranging from 1.0 μm to 2.6 μm. For comparison, the authors of publication [42] determined the range of surface roughness values of printed PEEK samples (immediately after printing) and after grinding and mechanical polishing; the test was performed on a contact profilometer. For samples after printing, the *Ra* value ranged from 0.067 µm to 0.613 µm. The values obtained meet the basic requirements for polymer endoprosthesis surfaces. However, for long-term implant components, for which osseointegration is important, the surface roughness values should be higher. Therefore, in the next stages of our research, PEEK samples will be modified by surface treatment with low-temperature plasma, which should ensure higher *Ra* and *Sa* values [43]. As demonstrated in article [42], surface modification using low-temperature plasma in an atmosphere of Ar, N_2_ and O_2_, carried out for a period of 15 min, allows for effective change in the surface properties of the material.

### 3.3. Mechanical Tests–Results and Discussion

#### 3.3.1. Tensile Strength Test–Results and Discussion

**Figure 10:**
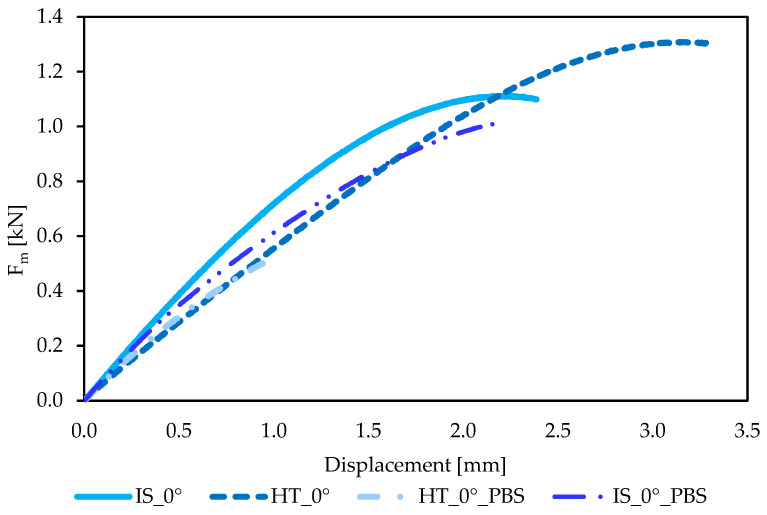
Example force–displacement curves for samples intended for tensile strength testing.

The graphs showing the relationship between force F_m_ and displacement obtained during the static tensile test of the samples are shown in Figure 10, while the average maximum force F_m_ [N] with standard deviation and the average tensile strength R_m_ [MPa] with standard deviation are presented in Table 5.

The data analysis revealed that the highest tensile strength (R_m_ = 78 MPa) was exhibited by samples subjected to the heating process, which was also confirmed by ANOVA statistical analysis (*p* < 0.05). The samples from the IS_0° group had lower tensile strength compared to the HT_0° samples; the difference was 16 MPa. The observed increase in the strength of the samples after heat treatment is due to the strengthening effect of the material. The greatest difference was observed for samples from the HT_0°_PBS group, for which the tensile strength value was significantly reduced compared to the maximum value of 78 MPa obtained for HT_0° samples. The value decreased to 52 MPa. The reduction in the strength properties of samples soaked in PBS solution may be related to the weakening of interlayer bonds formed during the FFF printing process. The authors of publication [42] showed that for samples printed at an angle of 45° (with an orientation of 0°), the tensile strength was approximately 60 MPa, which is consistent with the results of our own research. The similarity of the obtained results is also confirmed by the literature data presented by the authors [23], who showed that the heat treatment temperature plays a key role in shaping the mechanical properties of the material. After annealing, the tensile strength increased to 70.84 MPa, which is also confirmed by our own test results, where an increase in this value was observed for samples from the HT_0° group (from 62 MPa to 78 MPa).

#### 3.3.2. Compressive Strength Test–Results and Discussion

**Figure 11:**
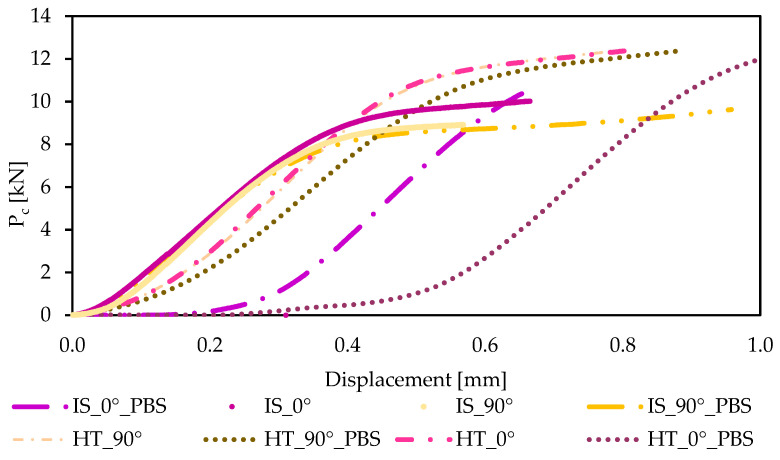
Example force–displacement curves for samples intended for compressive strength testing.

The graphs showing the relationship between P_c_ force and displacement obtained during the static compressive test are shown in Figure 11, while the average values of the compressive force P_c_ [N] together with the standard deviation and the average values of the compressive strength R_c_ together with the standard deviation [MPa] are presented in Table 6.

The results of the study indicate that the orientation of the samples for printing (at angles of 0° and 90°) and the heating process and soaking in PBS solution affect the compressive strength values obtained. For both analyzed sample orientations, the most favorable results were observed for samples subjected to annealing. The compressive strength of samples printed at an angle of 0° was 318 MPa, while for samples printed at an angle of 90°, the value was 350 MPa. Samples soaked in PBS solution for 28 days did not show such significant changes in compressive strength as observed in the static tensile test. The compressive strength values for samples printed at an angle of 0° range from 300 to 305 MPa, while for samples printed at an angle of 90° they range from 304 to 310 MPa (*p* < 0.05). The lowest compressive strength values were recorded for samples in their initial state. For samples printed at an angle of 0°, the difference compared to the highest compressive strength value obtained was 69 MPa. An even greater difference was observed for samples printed at an orientation of 90°, where this value decreased by 111 MPa. In publication [44], the authors also conducted compressive strength tests on cuboid specimens. However, static compression tests are most often performed on cylindrical specimens. Based on the results obtained, it can be observed that the maximum stress reached a value of approximately 120 MPa. In the case of our own test results, the lowest value was recorded for samples from the IS_90° group, for which the compressive strength R_c_ was 239 MPa. The values obtained for samples printed in the 0° orientation were very similar to each other, with a difference of approximately 10 MPa between the samples in their initial state, printed in the opposite orientation to the printed samples. At the same time, it should be emphasized that among all the strength tests performed, the highest values were recorded during the static compression test, which plays an important role in assessing the suitability of materials for long-term orthopedic implants.

#### 3.3.3. Bending Strength Test–Results and Discussion

**Figure 12:**
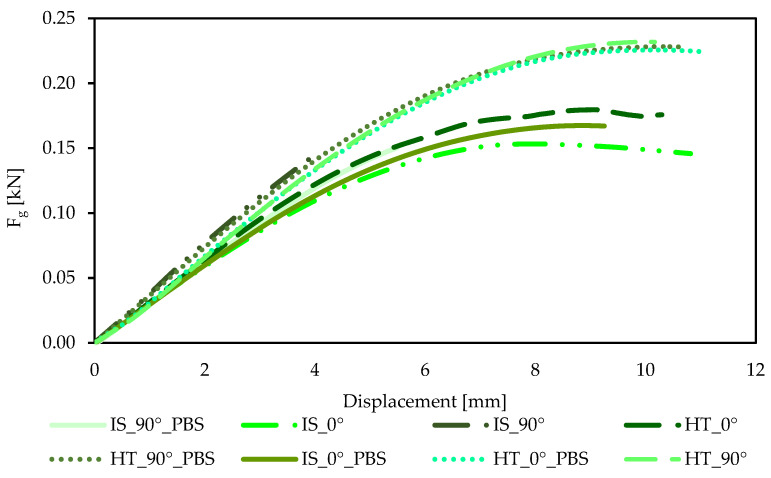
Example force–displacement curves for samples intended for bending strength testing.

The graphs showing the relationship between force F_g_ and displacement for the static bending test are shown in Figure 12, while the average bending force F_g_ [N] with standard deviation and the average bending strength R_g_ with standard deviation [MPa] are also shown. In addition, the deflection arrow value *f* [mm] was determined, as shown in Table 7.

As in the case of samples subjected to static tensile and compressive tests, the most favorable strength properties were obtained for samples after the annealing process, regardless of the print orientation. However, in the case of samples subject to heat treatment at an angle of 0°, an additional effect of soaking in PBS solution was observed, which resulted in an increase in flexural strength R_g_ from 36 MPa to 60 MPa. For samples printed in the same orientation and subjected only to heat treatment, the flexural strength was 46 MPa. The highest deflection value was recorded for samples from the HT_0°_PBS group, indicating that the combination of heat treatment and prolonged immersion in the PBS environment led to an increase in the material’s susceptibility to deformation while maintaining high flexural strength. The samples in their initial state after soaking in PBS solution (IS_0°_PBS) also exhibited higher flexural strength compared to the samples in their initial state without soaking, with a difference of 11 MPa. For samples printed at a 90° orientation, the bending strength values obtained were similar. Samples without heat treatment had a strength of approximately 40 MPa, while annealing samples achieved values of around 60 MPa (*p* < 0.05). Similarly to samples printed at an angle of 0°, the highest deflection arrow value was recorded for samples subjected to both the heating process and soaking in PBS solution. The authors of publication [23] also pointed out the significant impact of interlayer bonding quality, which affects the strength of the material. Samples printed in a horizontal orientation (0°) are characterized by weaker interlayer bonding, which results from lower adhesion between successive layers compared to stronger fiber bonds within a single layer. This confirms the higher flexural strength results of samples printed at an angle of 90°. In addition, the authors [45] observed that the thickness of the layer between adjacent filament layers has a significant impact on the strength properties obtained. For a layer thickness of 0.20 mm, the flexural strength reached 52.1 MPa, which may explain the lower R_g_ values of the samples in their initial state, printed in both orientations. This observation is from our own research. The differences in the results obtained when using a layer thickness of 0.15 mm, the flexural strength values in the static test were 36 MPa for samples from the IS_0° group and 41 MPa for samples from the IS_90° group, respectively.

#### 3.3.4. Hardness Measurement–Results and Discussion

**Figure 13:**
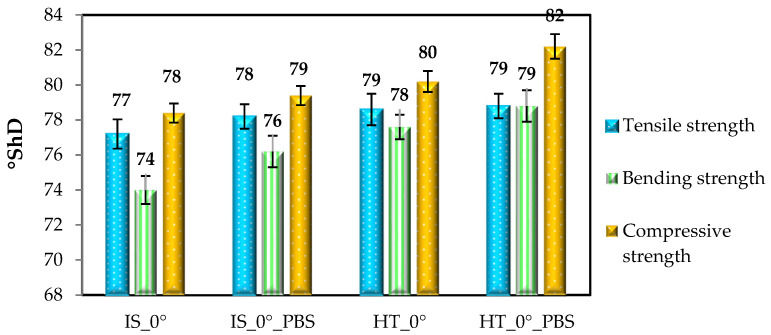
Shore D hardness values for samples arranged for printing at an angle of 0°. Error bars represent standard deviation.

**Figure 14:**
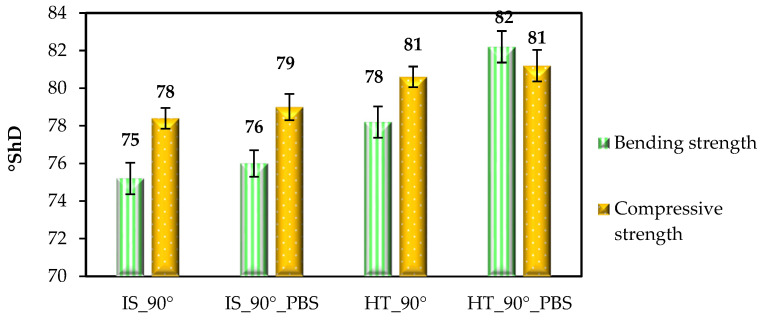
Shore D hardness values for samples arranged for printing at an angle of 90°. Error bars represent standard deviation.

The Shore D hardness results are shown in Figure 13 and Figure 14.

Analysis of the results of tests on samples printed for static tests—tensile, compressive, and bending—showed that the highest hardness values (already in the initial state) were obtained for samples intended for static bending testing. In this case, the hardness values ranged from 78 to 82 °ShD. As in other strength tests, the most favorable results were observed for samples subjected to heat treatment, while in the case of hardness tests, the highest values were obtained for samples from the HT_0°_PBS and HT_90°_PBS groups, which was confirmed by ANOVA statistical analysis (*p* < 0.05). For samples intended for static tensile testing, the hardness values obtained ranged from 77 to 79 °ShD. In the case of samples for static bending testing (printed in 0° orientation), the hardness values ranged from 74 to 79 °ShD. The lowest value (74 °ShD) was recorded for samples from the IS_0° group, while the highest value (79 °ShD) was recorded for samples that were annealing and additionally soaked in PBS solution. Samples printed in a 90° orientation showed only slightly higher hardness values compared to samples printed in a 0° orientation. For example, for samples intended for static bending testing, the hardness value in the initial state was 74 °ShD for the IS_0° group and 75 °ShD for the IS_90° group, which indicates a difference of 1 °ShD. As in other cases, the highest hardness values were obtained for samples from the HT_90° and HT_90°_PBS groups. The results of tests for samples from the 0° group, for which no significant differences in hardness values were found, are confirmed by the authors’ tests [42], which show that the strength properties of the material are influenced not only by the orientation of the samples relative to the working platform, but also by the printing angle itself. For samples printed at an angle of 45° (in the 0° position), the average hardness value was slightly above 80 °ShD.

### 3.4. Macroscopic Observations–Results and Discussion

**Figure 15:**
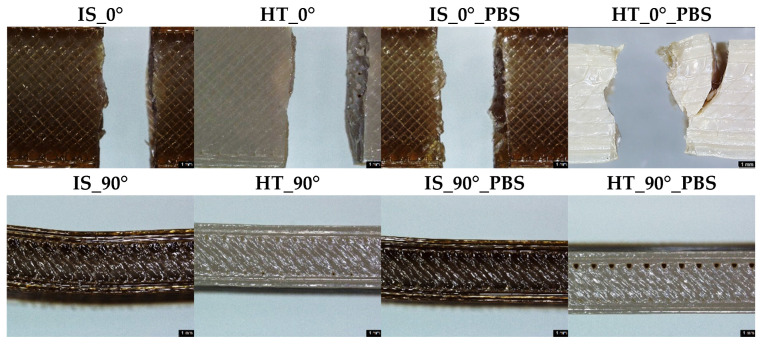
Macroscopic observations at 70× magnification; scale bar set at 1 mm.

Sample images of surfaces for samples after bending strength tests, printed in 0° and 90° orientation, are shown in Figure 15.

**Figure 16:**
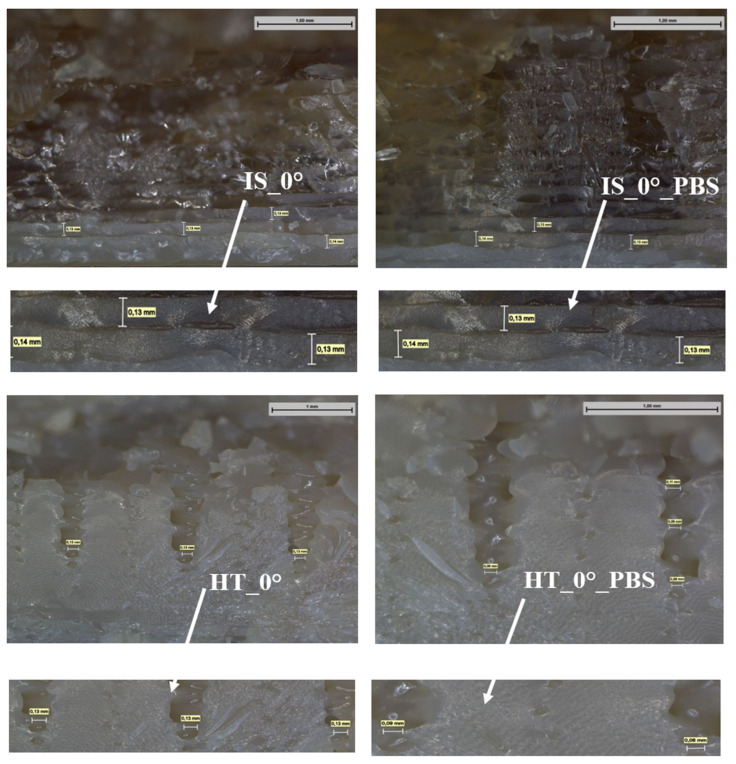
Distances between adjacent filament layers at 250× magnification.

Measurements of the distance between adjacent filament layers of the samples after static tensile testing are shown in Figure 16.

Based on macroscopic observations, for samples that were not annealing (IS_0° and IS_0°_PBS), the distances between individual filament layers were approximately 0.15 mm. However, for samples after the heating process, these distances have decreased, which is the result of material shrinkage during sample annealing. The values obtained range from 0.09 mm to 0.13 mm. Furthermore, in the case of tensile strength test results, the highest strength was recorded for samples from the HT_0° group, which may confirm that the resulting shrinkage of the material caused a reduction in the distance between the layers, which improved the strength properties of the samples.

### 3.5. Computed Tomography–Results and Discussion

**Figure 17:**
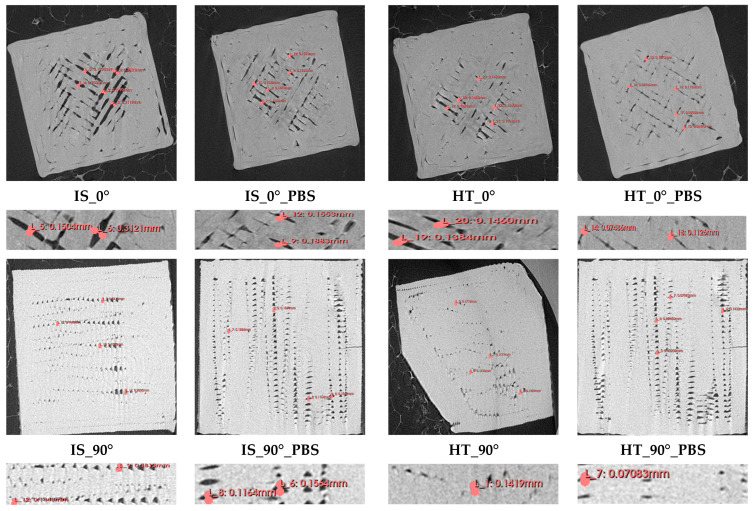
Microtomography images for static compression testing.

The CT images are shown in Figure 17, while the results of measurements of the distance between adjacent filament layers are presented in Table 8.

The results of the study indicate that the distances between adjacent filament layers for both sample groups (0° and 90°) are more accurate in computed tomography assessments than in measurements performed with a digital microscope. However, it should be noted that only samples intended for static compressive testing were selected for tomographic testing, due to the limited testing field of the device. The distance between layers set during the printing process was 0.15 mm. Values higher than the nominal values may indicate the incomplete or incorrect melting of successive filament layers during the manufacturing process. In addition, it was observed that in the case of samples printed in a 90° orientation, the values obtained showed greater consistency with the parameters set during printing. For samples from both analyzed groups, it was found that soaking in PBS solution for 28 days led to a reduction in the distance between the filament layers. Additionally, based on the results of the surface topography analysis, it was observed that the highest value of the surface roughness parameter *Sa* was recorded for samples from the HT_0°_PBS group. These results are confirmed by publication [23], in which, as indicated in subsection “3.3.3. Bending strength test,” samples printed in a horizontal orientation (0°) are characterized by weaker interlayer bonding.

## 4. Conclusions

Based on the research conducted and the results obtained, the following conclusions can be drawn:

- The most advantageous arrangement of samples in the FFF printing process was found to be a 90° orientation, which yielded the highest strength properties in both bending and compression tests.
- The use of the heat treatment processes each time led to an increase in the strength properties of the tested material, indicating the important role of this stage in the process of manufacturing samples intended for testing long-term orthopedic implants.
- Based on the results of other authors, it has also been confirmed that the orientation of the samples has a direct impact on the quality of interlayer bonds, which is less favorable for samples arranged at an angle of 0°.
- A notable decrease in strength from 78 to 26 MPa is evident when comparing the HT_0° and HT_0°_PBS groups. This decline is attributable to the combined effect of heat treatment and PBS soaking, rather than to orientation.
- In the future, tests are planned to be carried out during the soaking of samples for 3, 7, 10, 14, 21, 28, 42, and 56 days (as done by the authors of the publication [36]). The aim of such a study will be to conduct a comprehensive analysis of the stability of the material in conditions simulating the human body.
- The results obtained serve as a reference point for further research on the optimization of printing parameters, particularly FFF for orthopedic implants. Subsequent studies would also aim to change the ‘layer height’ and printing speed, which was also included in the review article [9] based on data from other authors’ publications. To demonstrate the differences, identical tests would be carried out to determine the differences in strength. In subsequent stages of the research, it is also planned to extend the analysis to include microstructure assessment and physicochemical testing. The aim of this research will be to determine the optimal surface treatment methods for effectively modifying the strength properties of the material and the physicochemical properties of the surface.

## Figures and Tables

**Table 1:** Mechanical properties of biomaterials [14,15,16,17,18,19,20,21].

Material	Yield Point R_p0.2_ [MPa]	Tensile Strength R_m_ [MPa]	Extension A [%]	Young’s Modulus E [MPa]
Ti6Al4V	795	860	10	112,000
Ti6Al7Nb	800	900	10	110,000
PEEK	116	116	15	4400

**Table 2:** Printing parameters for polyetheretherketone samples.

Fill Rate [%]	Angle Fills [°]	Layer Height [mm]	Temperature of the Modeling Material [°C]	Printing Time [h]
100	45	0.15	420	16.25

**Table 3:** Division of samples for testing.

Designation	Description
IS_0°	Initial state—samples positioned in the 0°/90° orientation
IS_90°	
IS_0°_PBS	Soaking in PBS solution for 28 days at 37 °C
IS_90°_PBS	
HT_0°	Heat treatment for 16 h at 156 °C
HT_90°	
HT_0°_PBS	Soaking in PBS solution for 28 days at 37 °C
HT_90°_PBS	

**Table 4:** Crystallinity calculated using XRD method for all samples.

Sample Name	Crystallinity [%]
IS_0	30
IS_0_PBS	32
IS_90	0
IS_90_PBS	0
HT_0	36
HT_0_PBS	38
HT_90	35
HT_90_PBS	38

**Table 5:** Results for samples after static tensile testing.

Samples	F_m_ [N]	R_m_ [MPa]
IS_0°	1133 ± 76	62 ± 6
IS_0°_PBS	1033 ± 62	57 ± 3
HT_0°	1380 ± 140	78 ± 4
HT_0°_PBS	481 ± 140	26 ± 7

**Table 6:** Results for samples after static compressive test.

Samples	P_c_ [N]	R_c_ [MPa]
IS_0°	945 ± 39	249 ± 15
IS_0°_PBS	1195 ± 69	305 ± 17
HT_0°	1239 ± 30	318 ± 8
HT_0°_PBS	1202 ± 20	300 ± 5
IS_90°	932 ± 51	239 ± 10
IS_90°_PBS	1174 ± 39	304 ± 9
HT_90°	1317 ± 65	350 ± 27
HT_90°_PBS	1198 ± 30	310 ± 15

**Table 7:** Results for samples after static bending test and deflection value.

Samples	F_g_ [N]	R_g_ [MPa]	f [mm]
IS_0°	142 ± 20	36 ± 4	7.8 ± 0.1
IS_0°_PBS	176 ± 3	47 ± 1	8.5 ± 0.1
HT_0°	182 ± 57	46 ± 1	8.9 ± 0.1
HT_0°_PBS	233 ± 4	60 ± 1	10.0 ± 0.1
IS_90°	163 ± 2	41 ± 1	4.7 ± 0.1
IS_90°_PBS	164 ± 3	42 ± 1	6.3 ± 0.1
HT_90°	233 ± 2	59 ± 1	9.7 ± 0.1
HT_90°_PBS	230 ± 3	59 ± 1	9.9 ± 0.1

**Table 8:** Distances between adjacent filament layers-CT.

Samples	Distances Between Layers [mm]			
*Measurement*	*1*	*2*	*3*	*4*
**IS_0°**	0.15	0.25	0.31	0.20
**IS_0°_PBS**	0.19	0.14	0.13	0.15
**HT_0°**	0.14	0.15	0.13	0.15
**HT_0°_PBS**	0.11	0.07	0.08	0.08
**IS_90°**	0.18	0.14	0.15	0.20
**IS_90°_PBS**	0.14	0.12	0.16	0.12
**HT_90°**	0.17	0.14	0.13	0.18
**HT_90°_PBS**	0.07	0.09	0.09	0.14

## Data Availability

The original contributions presented in this study are included in the article. Further inquiries can be directed to the corresponding author.
